# Case Report: Novel biallelic moderately damaging variants in *RTTN* in a patient with cerebellar dysplasia

**DOI:** 10.3389/fped.2023.1326552

**Published:** 2023-12-21

**Authors:** Ferruccio Romano, Elisabetta Amadori, Francesca Madia, Mariasavina Severino, Valeria Capra, Renata Rizzo, Rita Barone, Beatrice Corradi, Luca Maragliano, Mohammad Sadegh Shams Nosrati, Antonio Falace, Pasquale Striano, Federico Zara, Marcello Scala

**Affiliations:** ^1^ Genomics and Clinical Genetics Unit, IRCCS Istituto Giannina Gaslini, Genoa, Italy; ^2^ Department of Neurosciences, Rehabilitation, Ophthalmology, Genetics, Maternal and Child Health, Università Degli Studi di Genova, Genoa, Italy; ^3^ Child Neuropsichiatry Unit, IRCCS Istituto Giannina Gaslini, Genoa, Italy; ^4^ Medical Genetics Unit, IRCCS Istituto Giannina Gaslini, Genoa, Italy; ^5^ Neuroradiology Unit, IRCCS Istituto Giannina Gaslini, Genoa, Italy; ^6^ Child Neuropsychiatry Unit, Department of Clinical and Experimental Medicine, University of Catania, Catania, Italy; ^7^ Department of Experimental Medicine, University of Genova, Genova, Italy; ^8^ Center for Synaptic Neuroscience and Technology, Istituto Italiano di Tecnologia, Genova, Italy; ^9^ Department of Life and Environmental Sciences, Polytechnic University of Marche, Ancona, Italy; ^10^ Pediatric Neurology and Muscular Diseases Unit, IRCCS, Genoa, Italy

**Keywords:** *RTTN*, microcephaly, cognitive impairment, seizures, cerebellar dysplasia, multiple arachnoid cysts

## Abstract

Rotatin, encoded by the *RTTN* gene, is a centrosomal protein with multiple, emerging functions, including left-right specification, ciliogenesis, and neuronal migration. Recessive variants in *RTTN* are associated with a neurodevelopmental disorder with microcephaly and malformations of cortical development known as “Microcephaly, short stature, and polymicrogyria with seizures” (MSSP, MIM #614833). Affected individuals show a wide spectrum of clinical manifestations like intellectual disability, poor/absent speech, short stature, microcephaly, and congenital malformations. Here, we report a subject showing a distinctive neuroradiological phenotype and harboring novel biallelic variants in *RTTN*: the c.5500A>G, p.(Asn1834Asp), (dbSNP: rs200169343, ClinVar ID:1438510) and c.19A>G, p.(Ile7Val), (dbSNP: rs201165599, ClinVar ID:1905275) variants. In particular brain magnetic resonance imaging (MRI) showed a peculiar pattern, with cerebellar hypo-dysplasia, and multiple arachnoid cysts in the lateral cerebello-medullary cisterns, in addition to left Meckel cave. Thus, we compare his phenotypic features with current literature, speculating a possible role of newly identified *RTTN* variants in his clinical picture, and supporting a relevant variability in this emerging condition.

## Introduction

The *RTTN* gene (MIM × 610436) is located on chromosome 18 (18q22.2 region) and encodes a brain-enriched protein known as Rotatin, that plays crucial roles in brain development. In particular, Rotatin is involved in the early developmental processes of left-right (L-R) specification and axial rotation (1). More recently, Rotatin was found to be relevant to primary ciliogenesis, sonic hedgehog (SHH) signaling, and neuronal migration (2). Additionally, *RTTN* was suggested to regulate neuronal differentiation, centrosome biogenesis, and cell growth and duplication (2). Cilia formation and centrosome/centriole biogenesis are in fact considered overlapping and interdependent processes (3, 4). Rotatin is directly implicated in the regulation of different phases of the cell cycle and mutant cells show severe mitotic failure, leading to aneuploidy and apoptosis (5). However, several functional aspects of Rotatin remain poorly understood.

Biallelic variants in *RTTN* are associated with a neurodevelopmental condition characterized by microcephaly, epilepsy, and brain developmental abnormalities. The variety of cellular processes regulated by Rotatin partly explains the heterogeneity of clinical manifestations observed in *RTTN* patients, especially cerebral malformations, related to pathological proliferation and migration defects (6). Recently, a thorough revision of literature by Vandervore, including all reported cases by 2019, highlighted the “core features”, consisting of intellectual disability, poor/absent speech, short stature and variable cerebral malformations. These include congenital or secondary microcephaly, lissencephaly, gyration anomalies, periventricular heterotopia, and interhemispheric arachnoid cysts. The cortical malformations are often more evident in the frontal areas, suggesting elective underdevelopment of the frontal lobes (7). Among other clinical manifestations, seizures are described as well as other congenital malformations, especially involving the eye (microphtalmia, orbital abnormalities, and optic nerve hypoplasia), the heart, the kidney (pyelocaliectasis, renal ectopy, or agenesis), and/or the urogenital system (6, 8–10).

In this study we report a patient with novel biallelic *RTTN* variants with predicted moderate pathogenic effect, presenting with a milder clinical phenotype characterized by cognitive impairment and peculiar neuroimaging abnormalities, including cerebellar dysplasia.

## Methods

### Patient enrollment and clinical assessment

The study was conducted in accordance with the Declaration of Helsinki and approved by the local Institutional Ethics Committees. The patient was enrolled at Istituto Giannina Gaslini, Genoa, and clinically evaluated by pediatric geneticists and neurologists. Informed consent was obtained by the parents.

### Genetic investigation

For genetic testing, trio-WES was performed in the family on genomic DNA extracted from peripheral blood. Agilent Sure Select QXT Clinical Research Exome (Agilent Technologies, Santa Clara, CA, USA) was used and Sequencing data were processed with in-house software for the GATK Best Practices pipeline for WES variant analysis execution. After filtering for allele frequency (≤0.01% in public databases, including GnomAD v2.1.1; https://gnomad.broadinstitute.org/), candidate variants were screened according to family segregation, conservation (GERP score), predicted impact on protein function through *in silico* tools (including SIFT, PolyPhen-2, Mutation Taster), and presence in clinical databases (ClinVar) (11). The variants were eventually validated through Sanger sequencing and candidate variants were classified according to the American College of Medical Genetics and Genomics (ACMG) criteria (12).

### Protein modeling

**Figure 1:**
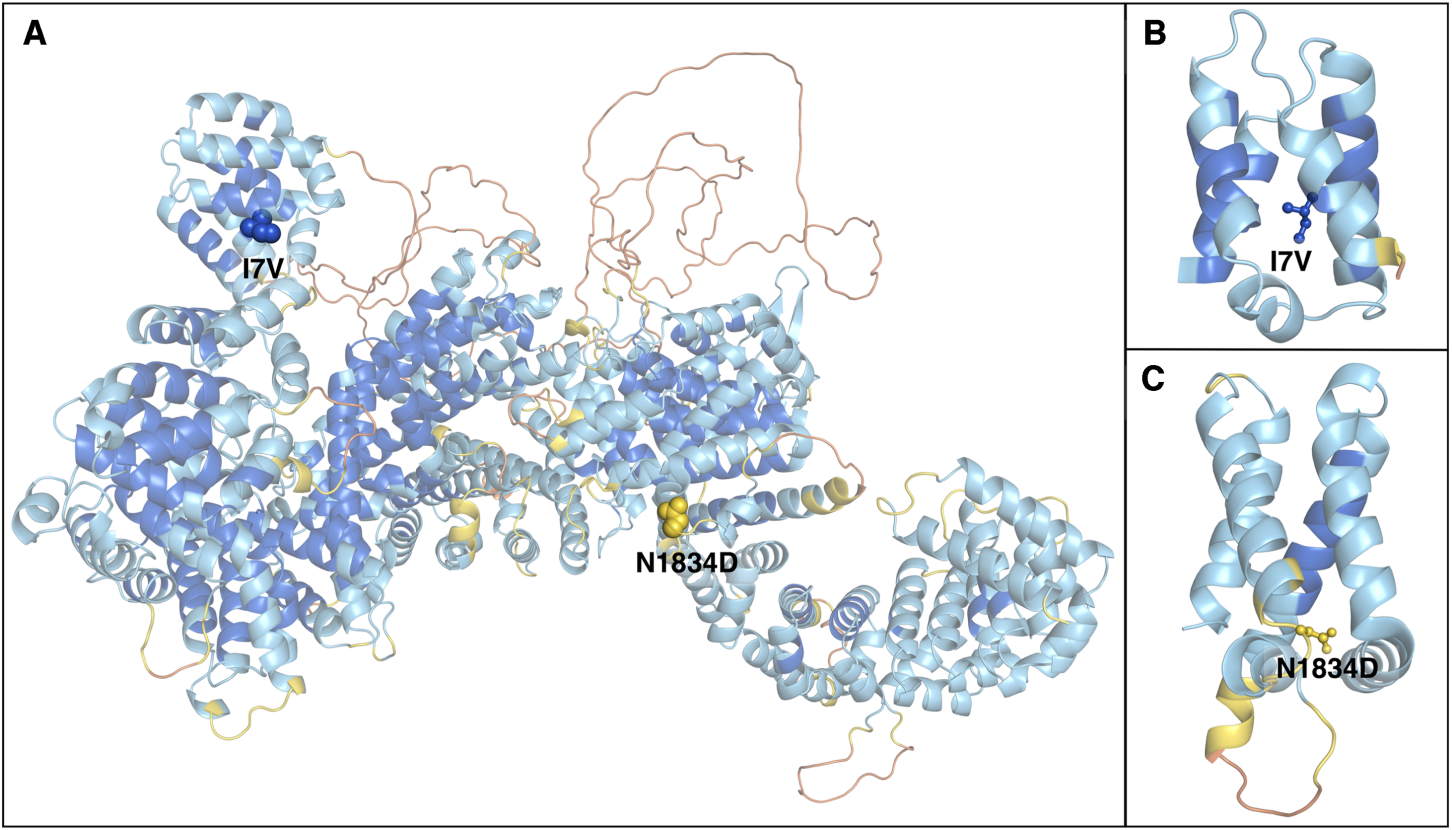
Structural model of the RTTN protein. (**A**) Alpha Fold predicted model of human rotatin. The protein is represented as cartoons and coloured according to the pLDDT confidence score, from orange (pLDDT < 50) to blue (pLDDT > 90). Mutated residues are represented as spheres and labeled. (**B,C**) enlarged views of the domains containing the mutations, which are represented as balls and sticks.

**Figure 2:**
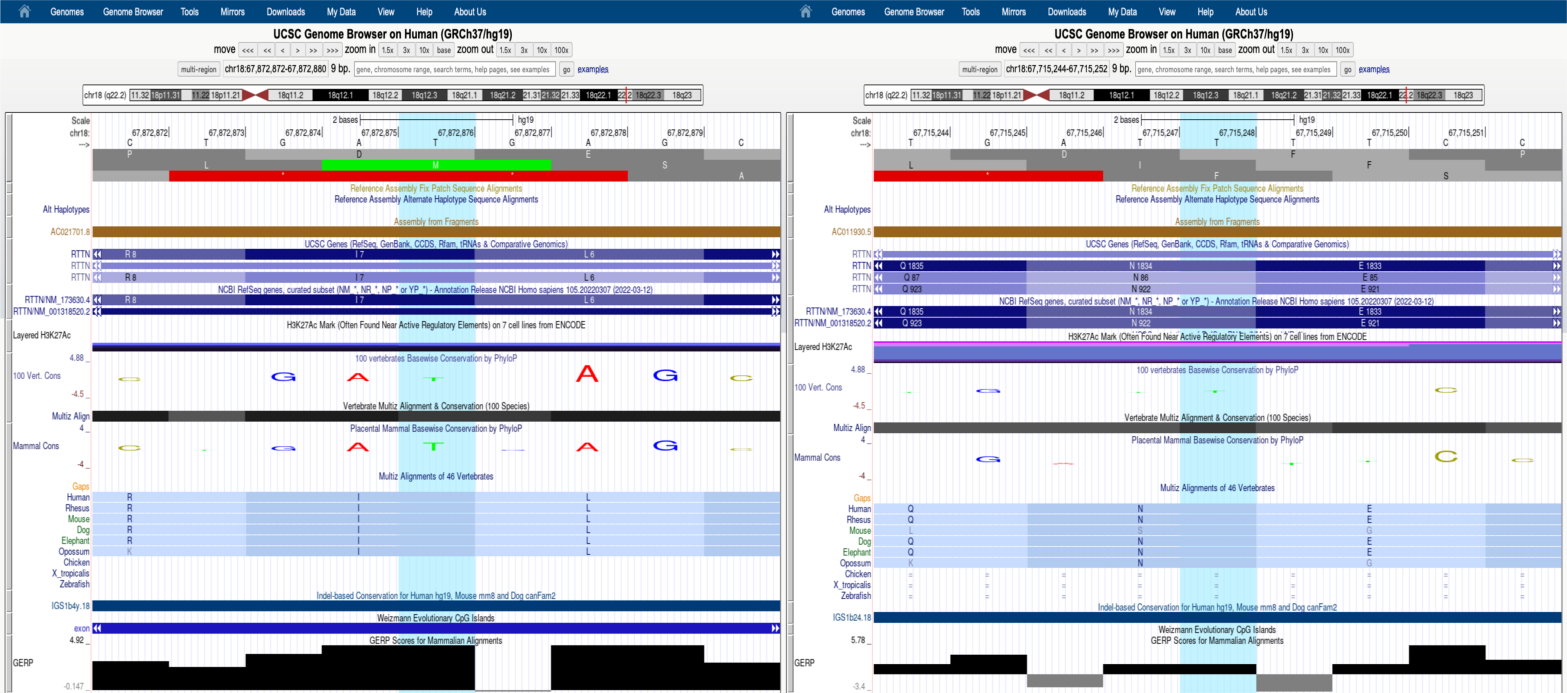
Extract from UCSC browser (hg19) showing the conservation of the residues affected by the variants harbored by our patient (highlighted in light blue) in different species and the relative GERP scores.

To better characterize the impact of the identified RTTN variants on protein structure, we introduced them in a 3D model of the WT molecule (Figure 1). The predicted structural model of wild-type (WT) human RTTN was retrieved from the AlphaFold Protein Structure Database (entry AF-Q86VV8-F1-model_v4) (13). The protein comprises 2,226 residues arranged in nearly all-helical domains, with the first 180 residues separated from the rest of the chain (Figure 2A). The model quality score (the predicted local-distance difference test, pLDDT) is higher than 70 (high confidence) for almost all the secondary structure elements and below 50 (low confidence) only for limited disordered regions and interhelical loops.

## Results

### Clinical description

This is the first 8-year-old boy of non-consanguineous parents of Italian ancestry. His mother is affected by juvenile myoclonic epilepsy since age 17 years and hypothyroidism. His father underwent surgical resection to treat focal epilepsy due to left temporal cortical dysplasia. Pregnancy was characterized by threatened abortion at 4 months. Due to placenta praevia a caesarian section was performed at 37 weeks of gestation. At birth, he only showed mild global hypotonia. Growth parameters at birth are not available but reported within the normal range. Ultrasonography revealed patent ductus arteriosus and enlarged renal pelvis. During the first months of life, the child showed signs of psychomotor delay: head and trunk control was achieved at 8 months, autonomous walking at 17 months, and the first words were pronounced at 18 months, with slow speech progression. Axial hypotonia persisted. At 4 months, the patient began to experience daily absences with eye deviation, lasting a few minutes and with spontaneous resolution. Electroencephalograms (EEGs) showed generalized high voltage epileptic abnormalities (worsened by sleep) in the frontal and left temporal regions. Valproic acid (15 mg/kg/day) was started, with poor clinical response. During the following years, behavioral issues emerged, namely attention deficit hyperactivity disorder (ADHD), severe speech impairment, motor clumsiness, and aggressiveness. Wechsler Preschool and Primary Scale of Intelligence (WPPSI-IV) revealed global psychomotor delay, especially in the verbal/communicative area. Autism Diagnostic Observation Schedule (ADOS-2) led to a diagnosis of autism spectrum disorder (ASD). At last clinical evaluation (age 8 years and 3 months) growth parameters were the following: height 138 cm (75th–90th percentile), weight 42.2 kg (>97th percentile) and head circumference 51 cm (10th percentile).

### Neuroimaging analysis

**Figure 3:**
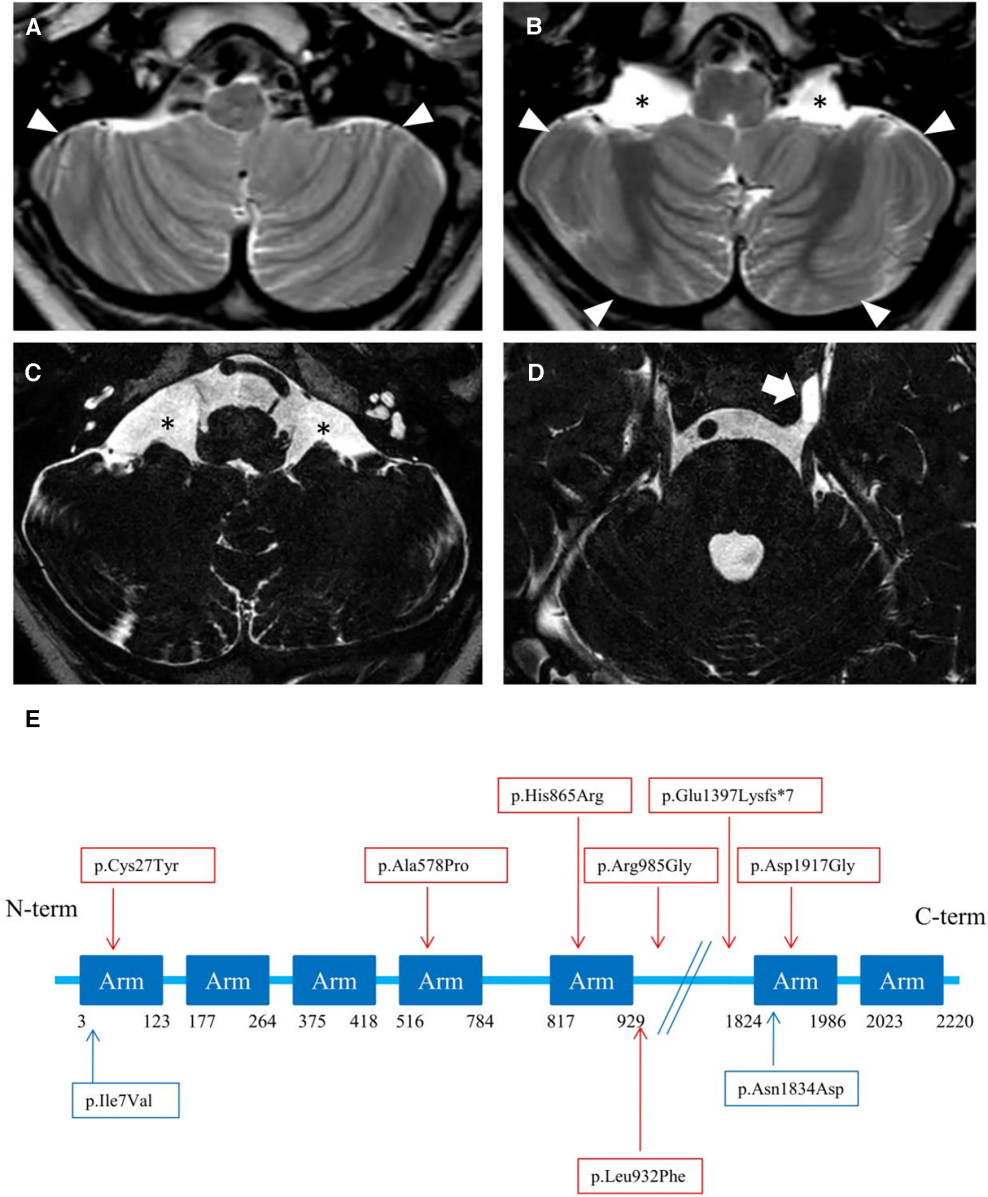
(**A–D**) neuroimaging of the patient. Brain MRI with axial T2-weighted images (**A, B**) and high-resolution heavily T2-weighted DRIVE images (**C, D**) reveal abnormal foliation pattern in the inferior portions of smaller cerebellar hemispheres (arrowheads). There are multiple arachnoid cysts localized in the lateral cerebello-medullary cisterns (asterisks) and left Meckel cave (thick arrow). (**E**) Linear structure of Rotatin protein showing Armadillo type fold (Arm aminoacids) repeat-rich areas mediating protein-protein interaction, modified from Cavallin et al. (7). The variants identified in the patient of this study are highlighted in blue, others are reported in red.

Brain MRI at 3 years and 7 months of age showed cerebellar hypo-dysplasia characterized by abnormal foliar pattern and mildly reduced hemispheric volume, associated with multiple arachnoid cysts in the lateral cerebello-medullary cisterns and left Meckel cave (Figure 3 and Supplementary Figure S1). In addition, there was a developmental venous anomaly in the left posterior temporal region associated with an abnormal cortical infolding and white matter gliosis (Supplementary Figure S2). A small ecchordosis physaliphora was noted at the level of the clivus, while a cystic pineal gland with signs of internal bleeding was detected as incidental finding (Supplementary Figure S2).

### Genetic results

Genetic investigation included Comparative Genomic Hybridization (CGH)-array, analysis for Fragile-X syndrome and a multi-gene panel for epileptic encephalopathies. All these tests yielded negative results. As a further step of investigation, whole exome sequencing (WES) was performed on DNA from blood's lymphocytes of the proband and his parents. We identified two compound heterozygous variants in the *RTTN* gene (NM_173630.4) in the patient: the paternally inherited c.5500A>G, p.(Asn1834Asp) variant (rs200169343, ClinVar ID 1438510), and the maternally-inherited c.19A>G, p.(Ile7Val) variant (rs201165599, ClinVar ID 1905275).

### Protein modeling

The modelling of the variants on the three-dimensional RTTN protein structure showed that the two mutated residues p.(Ile7Val), (I7V), and p.(Asn1834Asp), (N1834D) are located in the first helix of the outlying N-terminal and on a loop of a tightly packed region, respectively (Figure 1B,C). The impact of mutations on protein stability can be estimated by the difference between the unfolding free energy of the mutated and the WT protein (ΔΔG), with negative values indicating destabilization. We used a machine learning method, ACDC-NN (14), to calculate ΔΔG values for the two substitutions, obtaining −0.60 Kcal/mol and −0.17 Kcal/mol for I7V and N1834D, respectively, thus identifying both as destabilizing.

## Discussion

### Typical and atypical clinical features

We report a subject harboring compound heterozygous variants in *RTTN*, presenting with both typical and atypical clinical/neuroradiological features. In addition to the principal neurodevelopmental features of MSSP, our patient suffers from untreatable epilepsy and persistent hypotonia, which are observed in about 17% of cases (6). In line with previously described subjects, our patient presents with behavioral issues, including ADHD, ASD, and aggressiveness. He was also diagnosed with a calico-pyelic dilatation, reported in almost 22% of patients (6). No additional malformation could be detected. Of note, while poor growth and microcephaly are considered seminal features of MSSP, we did not observe them in our patient. The clinical phenotype of MSSP, however, is particularly heterogeneous (Supplementary Table S1) (7) and it is known that microcephaly may be lacking in affected individuals: about 81% of the individuals are microcephalic at birth, and in total 86% have secondary microcephaly, which means that normal head circumference at birth is not an exclusion criterion for *RTTN* mutations (6).

### Typical and atypical neuroradiological features

The cerebral malformations identified in our patient were less severe than other *RTTN* cases, which are characterized by microcephaly, simplified gyration, lissencephaly/pachygyria, polymicrogyria, nodular heterotopias, midline defects, and cerebellar hypoplasia (6). Indeed, we noticed a peculiar form of cerebellar hypo-dysplasia characterized by bilateral abnormal foliation at the level of the inferior portions of the cerebellar hemispheres with mildly reduced cerebellar volume. The cerebellar cortical pattern has not been described in detail in *RTTN* subjects with cerebellar hypoplasia. The possible role of *RTTN* in cerebellar development is not elucidated to date, and cannot be excluded. Vandervore and colleagues reported the presence of (ponto) cerebellar hypoplasia in 30% of patients (2, 3, 6, 8, 9). Thus, the peculiar foliar arrangement observed in our patient might reflect a rotatin defect. No additional malformation of cortical development was detectable, except for a focal cortical infolding associated with a developmental venous anomaly. Based on current knowledge, we can only speculate that isolated cerebellar dysplasia, in absence of other malformative features, may suggest the possibility of a milder spectrum than expected in MSSP.

Arachnoid cysts in subjects with *RTTN* variants have been described in several locations, including the posterior interhemispheric region, quadrigeminal cistern, and anterior temporal regions. Large interhemispheric cysts were sometimes associated with severe brain disruption, like the hydrocephaly of *Rttn*-/- knockout mice (1). Finally, two incidental findings were detected, including an ecchordosis physaliphora, a congenital benign hamartomatous lesion originating from nodal cord remnants (15), and a pineal cyst complicated by hemorrhage.

### Hypotheses on the phenotypic role of *RTTN* variants

In our patient, WES analysis led to the identification of two distinct missense variants that the patient harbored in compound heterozygous state. The paternal c.5500A>G, p.(Asn1834Asp) variant is rare in gnomAD (allele frequency 0.0000882) and never reported in homozygous state in healthy individuals. It is classified as a variant of uncertain significance (VUS) with the following parameters: PM2 and BP4. It affects a conserved residue (GERP = 5.78, Figure 2) within the sixth Arm domain of the protein and is predicted to be only moderately damaging according to *in silico* tools (CADD score = 15.63; SIFT = 0.031; Polyphen-2 = 0.554). Similarly, the maternal c.19A>G, p.(Ile7Val) variant is rare in gnomAD (allele frequency 0.000104) and is never reported in homozygous state in healthy subjects. According to ACMG classification it is considered a VUS (PM2, BP4). This change affects a conserved residue (GERP score = 4.92, Figure 2) within the first Arm domain of the protein and is predicted to have a moderate pathogenic impact (CADD score = 20.9; SIFT = 0.598; Polyphen-2 = 0.023). No additional variants with potential pathogenic impact in other disease-related genes was identifiable and no potentially damaging inherited dominant variant was detected in the proband. Despite the conflicting in silico predictions, both variants are very rare in the general population and only reported in heterozygous state in healthy subjects. Furthermore, they affect conserved residues within functional domains of the RTTN protein and fall in close proximity to previously reported variants associated with MSSP (Figure 3). It is worth noticing that both variants are considered having a destabilizing effect, based on Alphafold protein modeling (Figure 1).

We cannot exclude that the milder phenotype of our patient might reflect a less deleterious effect on protein function of the underlying mutations. Thus, it is possible that the severity and complexity of the phenotype depends on the amount of residual functional rotatin (3). RTTN functions have not yet been fully explored and further studies are required to investigate this hypothesis.

### Limitations of the study

A limitation of our study is the unavailability of recent parental brain MRIs. Thus we cannot exclude the presence of subtle brain malformations in the parents. Other possible genetic factors segregating in the family have not been identified, but cannot be excluded. Another limitation is represented by the absence of functional studies, demonstrating a clear (though mild or moderate) effect of the identified variants.

### Genotype-phenotype correlations

The phenotypic features in association with biallelic *RTTN* mutations is heterogeneous, including a vast spectrum of clinical entities. The milder end of the clinical spectrum consists of a neurodevelopmental delay syndrome with mild growth deficiency and a polymicrogyria-like cortical malformation. At the severe end of the spectrum, there is microcephalic primordial dwarfism with different kinds of cortical malformations (6, 8). Extreme phenotypes are reported too, characterized by complex cerebral malformations, heart abnormalities, joint contractures and kidney defects leading, in some cases, to early death (8, 16). The differential diagnosis includes neurodevelopmental conditions with microcephaly and malformations of cortical development, such as the spectrum of complex disorders associated with RAC proteins dysfunction (17–19). Due to the limited number of patients with recessive *RTTN* mutations it is not clear if some of the sporadically described clinical features are part of the syndrome, or should be considered incidental findings. This is the case of hearing loss, duodenal atresia, and congenital joint contractures (20). More studies are needed to clarify these associations.

We cannot infer clear genotype-phenotype correlations in MSSP. A possible hypothesis is that the protein encoded by *RTTN* is large (2,226 aminoacids) and has many distinct protein-interacting domains, that act differently during the diverse phases of the cell cycle. Another possible explanation links the amount of *RTTN* mRNA expression with clinical severity (6). The role of possible modifier genes cannot be excluded. However, the severe phenotypic manifestation associated with several intronic variants suggest the importance of careful analysis of *RTTN* non-coding regions, especially in case of patients with a severe clinical picture and a single known *RTTN* variant (6). Moreover, the mechanisms by which *RTTN* is involved in cell cycle, neuronal migration and ciliogenesis is under debate, still making it difficult to understand how aberrant *RTTN* gene might result in such a wide range of clinical features. Further studies and clinical description of new cases will be helpful to better understand the complex interplay between mutated *RTTN* and clinical/neuroradiological issues and to infer a clearer genotype-phenotype association.

## Conclusion

We report a patient with novel compound heterozygous variants in *RTTN* gene presenting with both typical and atypical clinical and neuroradiological features. From a clinical point of view, the patient presents with MSSP signs like ID, ASD, ADHD, aggressiveness, epilepsy and pyelectasis. The neuroradiological findings observed in our patient are milder than the classical pattern reported in MSSP. Furthermore, our patient had normal growth parameters and head circumference, in contrast with most other *RTTN* individuals. In our case, there were persistent hypotonia and epilepsy, that are only described in a minority of *RTTN* patients. Overall, we compare our patient's phenotypic features with the ones reported in literature, discussing a possible involvement of newly identified *RTTN* biallelic variants in his milder clinical and neuroradiological picture. Further studies are needed to better understand the biologic functions of rotatin, and pathological consequences of its genetic mutations.

## Data Availability

The original contributions presented in the study are included in the article/**Supplementary Material**, further inquiries can be directed to the corresponding authors.

## Supplementary material

Supplementary Figure S1 Neuroimaging findings of cerebellar hypo-dysplasia. Brain MRI of the patient with axial T2-weighted images at the level of the cerebellum in a normal age-matched subject (**A**) and in the patient at 7.5 years of age (**B**). In the patient the volume of the cerebellar hemispheres is reduced and the foliation pattern is abnormal (empty arrows).

Supplementary Figure S2 Brain incidental findings of the patient. Brain MRI of the patient with axial susceptibility weighted (**A**), T1-weighted (**B**) and FLAIR (**C**) images demonstrating a developmental venous anomaly with the typical ‘caput medusae’ (curved arrows) associated with an abnormal cortical infolding (thin arrows) and signal alterations of the surrounding white matter (arrowheads). Sagittal high-resolution heavily T2-weighted DRIVE image (**D**), post-contrast T1-weighted image (**E**) and sagittal-reformatted CT scan (**F**) show a very small non-enhancing retroclival mass (thick arrows) associated with a bony clival defect, visible as a well-demarcated smoothly corticated region, without aggressive features (thin arrow). Note the cystic pineal gland complicated by internal bleeding (arrowheads).

Supplementary Table S1 Clinical Spectrum of phenotypes associated with RTTN variants. Reviewed and modified from Imran Naseer et al. (10). ND, not determined; M, male; F, female.
