# Supplementary material for: Case Report: Novel biallelic moderately damaging variants in RTTN in a patient with cerebellar dysplasia
Source: Front Pediatr. 2023 Dec 21;11:1326552. doi: 10.3389/fped.2023.1326552 (PMC10764497; doi:10.3389/fped.2023.1326552)
Supplement: Supplementary Table S1 — Clinical Spectrum of phenotypes associated with RTTN variants. Reviewed and modified from Imran Naseer et al. (10). ND, not determined; M, male; F, female. [file Datasheet1.pdf]

family	genotype	amino acidic change	state	origin	affected member	sex	microcephaly	short stature	psychomotor delay	seizures	kidney anomalies	joint contractures	reference
1.	c.19A>G; c.5500A>G	p.Ile7Val; p.Asn1834Asp	compound heterozygous	Italy	unique	M	no	no	yes	yes	pyelectasis	no	present study
2.	c.2796A>T	p.Leu932Phe	homozygous	Turkey	I	M	yes	yes	yes	yes	small kidney volume	no	kheradmand Kia et al., 2012
					II	M	yes	yes	yes	yes	ND	no	
					III	F	yes	yes	yes	yes	ND	no	
3.	c.80A>G	p.Cys27Tyr	homozygous	Turkey	unique	M	no	yes	yes	yes	no	no	kheradmand Kia et al., 2012
4.	c.2885+8A>G	p.Ser963*	homozygous	Yemen	I	M	yes	yes	yes	no	single kidney	no	Shamseldin et al., 2015
					II	M	yes	yes	yes	no	ectopic right kidney	no	
					III	M	yes	yes	yes	no	no	no	
5.	c.3190A>C	p.Lys1064Gln	homozygous	Saudi Arabia	unique	M	yes	yes	yes	no	ND	no	Shamseldin et al., 2015
6.	c.1732G>C; c.5750A>G	p.Ala578Pro; p.Asp1917Gly	compound heterozygous	Canada	I	M	yes	yes	death at 2 months	no	no	yes	Shamseldin et al., 2015
					II	M	yes	yes	death at 17 days	no	no	yes	
7.	c.2953A>G	p.Arg985Gly	homozygous	Morocco	I	M	yes	yes	yes	no	ND	no	Grandone et al., 2016
					II	F	yes	yes	yes	no	ND	no	
8.	c.190G>T; c.32-3C>T	p.Val64Phe; -	compound heterozygous	Europe	unique	M	yes	yes	death at 4 months	yes	no	yes	Wambach et al., 2018
9.	c.5225A>G; c.6038G>T	p.His1742Arg; p.Cys2013Phe	compound heterozygous	Saudi Arabia	unique	M	yes	yes	yes	yes	ND	no	Imran Naseer et al., 2021
